# Plasma Glycosaminoglycan Profiles in Systemic Sclerosis: Associations with MMP-3, MMP-10, TIMP-1, TIMP-2, and TGF-Beta

**DOI:** 10.1155/2020/6416514

**Published:** 2020-04-21

**Authors:** Kornelia Kuźnik-Trocha, Katarzyna Winsz-Szczotka, Katarzyna Komosińska-Vassev, Agnieszka Jura-Półtorak, Anna Kotulska-Kucharz, Eugeniusz J. Kucharz, Przemysław Kotyla, Krystyna Olczyk

**Affiliations:** ^1^Department of Clinical Chemistry and Laboratory Diagnostics, Faculty of Pharmaceutical Sciences in Sosnowiec, Medical University of Silesia, Katowice, Poland; ^2^Department of Internal Medicine, Rheumatology and Clinical Immunology, Faculty of Medical Sciences in Katowice, Medical University of Silesia, Katowice, Poland

## Abstract

The aim of the study was to determine whether plasma levels of total glycosaminoglycans (GAGs), matrix metalloproteinases (MMPs) (MMP-3, MMP-10), and their tissue inhibitors (TIMPs) (TIMP-1, TIMP-2) as well as transforming growth factor *β* (TGF-*β*) differ in the patients with systemic sclerosis (SSc) in relation to the healthy subjects. Plasma samples were obtained from 106 people (64 patients with SSc and 42 healthy individuals) and measured for MMP-3, MMP-10, TIMP-1, TIMP-2, and TGF-*β* levels using ELISA methods. GAGs isolated from plasma samples were quantified using a hexuronic acid assay. The plasma levels of total GAGs, TIMP-1, TIMP-2, and TGF-*β* were significantly higher, while MMP-3 was significantly decreased in SSc patients compared to the controls. We have revealed a significant correlation between plasma GAGs and TGF-*β* (*r* = –0.47) and TIMP-2 (*r* = 0.38), respectively. The results of this study revealed that remodeling of the extracellular matrix, reflected by quantitative changes in plasma glycosaminoglycans, occurs during systemic sclerosis. Thus, the alterations in GAG metabolism connected with SSc may lead to systemic changes in the properties of the connective tissue extracellular matrix.

## 1. Introduction

Systemic sclerosis (SSc) is an autoimmune disease of unknown etiology and pathogenesis that is characterized by microvasculopathy, immune system disturbances, and increased tissue deposition of collagen in the skin and internal organs, such as the lungs, gastrointestinal tract, heart, and kidneys [1–4]. It is believed that the reason for fibrosis in the course of SSc is not only the excessive biosynthesis of collagen by stimulated fibroblasts but also the increase in the number of other extracellular matrix (ECM) components, including proteoglycans (PGs) and their constituents, i.e., glycosaminoglycans (GAGs) [1–6]. Changes in PG/GAG metabolism, manifested by the accumulation of these macromolecules in tissues, depend on many factors, including IL-1, IL-4, platelet-derived growth factor (PDGF), insulin-like growth factor 1 (IGF-1), and transforming growth factor (TGF-*β*) [1, 3, 5, 7]. However, it has not been elucidated whether the accumulation of tissue PGs/GAGs is merely a manifestation of increased biosynthesis of these compounds or also their impaired degradation. The extracellular breakdown of PGs/GAGs, expressed by the plasma GAG level, takes place due to enzymatic processes, catalyzed by specific metalloproteinases and endoglycosases, as well as by the participation of nonenzymatic agents, i.e., reactive oxygen species [7]. It has been noticed that in the course of SSc there is an increased, both, in free radical and enzymatic cells, but only endoglycosidase catalyzed the degradation of the abovementioned matrix components [8, 9]. In contrast, the mode of action of metalloproteinases, including MMP-3 and MMP-10, i.e., enzymes degrading proteoglycan core proteins, is still not fully understood. It is suggested, however, that the overexpression of TGF-*β*, which is able to inhibit the expression of MMPs and induce the expression of their tissue inhibitors, may lead to a reduction in MMP activity [1, 3]. This, in turn, may contribute to the disturbance of the proteolytic-antiproteolytic balance in favor of the latter. Our study conducted on systemic sclerosis patients is an attempt at explaining these ambiguities. We decided to investigate the relation between the changes in heteropolysaccharide components of ECM and parameters which characterized an important proteolytic pathway affecting matrix remodeling, i.e., the concentration of matrix metalloproteinases (MMP-3 and MMP-10) and their tissue inhibitors (TIMP-1 and TIMP-2). To accomplish the main objective, we decided to quantify total GAG concentration in the blood, which reflects tissue alterations of PGs/GAGs, and to assess quantitatively MMP-3 and MMP-10 and their tissue inhibitors, i.e., TIMP-1 and TIMP-2, as well as TGF-*β* in the plasma of patients with systemic sclerosis, when compared to healthy individuals. An analysis of the correlation between the examined parameters and duration of the disease and the value of the Rodnan index was also conducted.

## 2. Materials and Methods

The study was carried out on 106 plasma samples obtained from 64 Polish patients with diffuse cutaneous systemic sclerosis (52 women and 12 men; mean age 54 years) and 42 age-matched and sex-matched healthy controls. Patients were classified as fulfilling the 2013 ACR/EULAR criteria for SSc [10]. Skin involvement was measured using the modified Rodnan skin score (mRss). The degree of skin thickness is measured in 17 body areas on a scale from 0 (normal) to 3 (severe), for a total score range of 0–51 [11]. The average value of mRss in diffuse cutaneous systemic sclerosis (dcSSc) patients was 22.03 ± 13.09 (mean ± SD). The mean disease duration was 4.40 ± 2.23 years. Duration was calculated from the moment of the onset of the first clear clinical manifestation of SSc (excluding Raynaud's phenomenon).

Laboratory workup included complete blood counts (platelets (218.23 ± 88.27 10^3^/*μ*L), red blood cells (4.29 ± 0.42 10^6^/*μ*L), white blood cells (7.44 ± 2.99 10^3^/*μ*L), hematocrit (37.48 ± 3.94%), hemoglobin (12.51 ± 1.45 g/dL), and erythrocyte sedimentation rate (ESR, 18.0 (13.0-29.0) mm/h (medians, quartile 1–quartile 3) as well as serum levels : fasting glucose (86.1 ± 12.79 mg/dL), total protein (6.31 ± 0.81 g/dL), C-reactive protein (CRP, 5.0 (4.0-8.0) mg/L (medians, quartile 1–quartile 3)), and *γ*-globulins (18.12 ± 5.66 g/L). Serological tests demonstrated the presence of antinuclear antibodies (ANA) and antibodies against topoisomerase I (Scl-70) in all SSc patients. Patients were treated with disease-modifying antirheumatic drugs, namely, methotrexate, corticosteroids, or cyclophosphamide.

Exclusion criteria were a history of alcohol abuse, chronic and acute diseases of the liver and kidney, diabetes mellitus, cancer, and other autoimmune diseases (so-called overlap syndromes).

The control material was plasma taken from 42 healthy individuals (34 women, 8 men). These persons had not been ill for the past five years, were not treated surgically, were not hospitalized, and were not treated pharmacologically during the course of the study. In addition, healthy individuals had basic laboratory tests in the last three months before the start of the planned study, including the level of leukocytes, erythrocytes, thrombocytes, hematocrit index, ESR values, and hemoglobin, as well as the evaluation of protein, glucose, total cholesterol, triglycerides, and alkaline phosphatase activity. The results of routine hematological and biochemical blood assays allowed the eliminatipon of subjects whose values of the assessed parameters differed from the reference values.

Blood from both healthy individuals and dcSSc patients was collected, after overnight fasting, into citrate-treated (extraction and determination of plasma GAGs) and heparin-treated (measurement of plasma MMP-3, MMP-10, TIMP-1, TIMP-2, and TGF-*β* levels) tubes.

Samples were gently mixed and centrifuged for 10 min at 2500 × g; then, the plasma was divided into portions and stored in aliquots at -80°C until the initiation of the study.

Informed consent was obtained from all participants according to the ethical guidelines of the Declaration of Helsinki. The study was carried out with the approval of the Local Ethical Committee of the Medical University of Silesia, Katowice, Poland. All participants gave their written informed consent. No conflicts of interest have occurred during implementation and completion of the study.

### 2.1. Extraction and Determination of Plasma Total GAGs

GAGs were isolated by the method of Volpi et al. [12] and Olczyk et al. [13]. GAGs were released from plasma PG core proteins by papain hydrolysis (24 h, 65°C) and alkali elimination (NaOH, 24 h, 40°C, and pH 9). From the obtained hydrolysates, protein breakdown products and nucleic acids were subsequently precipitated using a solution of trichloroacetic acid (TCA). The residues were discarded, and the GAGs were precipitated from the supernatant by adding three volumes of acetone (24 hours, at 4°C). The glycosaminoglycan sediments obtained as a result of centrifugation (25000 × g, 20 min, and 4°C) were dissolved in the potassium acetate solution. From the obtained solutions, glycans were reprecipitated with three volumes of ethanol and left for 12 hours at 4°C. Following centrifugation, precipitate was dissolved in H_2_O, and GAGs were isolated by precipitation after the addition of cetylpyridinium chloride (CPC). After incubation and centrifugation, GAGs precipitated by CPC were finally washed with C_2_H_5_OH containing NaCl and centrifuged once more. The supernatants were discarded and a final precipitate (isolated and cleared serum GAGs) was stored at -80°C until use for a biochemical analysis. The total amount of GAGs was determined by the hexuronic acid assay according to the Blumenkrantz and Asboe-Hansen [14] method, with analytical sensitivity of 0.5 mg/L and calibration range from 0.5 to 50 mg/L. The coefficient of intra-assay variation was less than 6%. This method does not measure the concentration of keratan sulfates, which do not contain hexuronic acids in their structure.

### 2.2. The Assay of the Concentration of MMP-3 and MMP-10 in Plasma Samples

MMP-3 and MMP-10 were quantitatively measured using a Human Quantikine Immunoassay (R&D Systems Inc., Minneapolis, USA) according to the manufacturer's protocol. The MMP quantikine kits detect total enzyme levels (pro and active forms). The samples for MMP-3 determination were diluted ten times before use, and those for MMP-10 determination were diluted twice. The results obtained with natural human MMP-3 and MMP-10 showed linear curves that were parallel to the standard curves obtained when using the recombinant quantikine kit standards, which indicates that quantikine kits can be used to determine relative levels of natural human MMP-3 and MMP-10. The results from these assays were determined from the standard curves in which the known concentrations of various standards were plotted against their optical density values. The mean minimum detectable concentration was 0.009 ng/mL for MMP-3 and 4.13 pg/mL for MMP-10. In both tests, maximum intra-assay CVs were less than 5%.

### 2.3. The Assay of the Concentration of TIMP-1 and TIMP-2 in Plasma Samples

TIMP-1 and TIMP-2 concentrations in plasma samples were measured using the Quantitative Human TIMP-1 and TIMP-2 Immunoassays from R&D Systems. The minimal detectable concentrations were 0.08 ng/mL for TIMP-1 and 0.01 ng/mL for TIMP-2. These assays measured both free and bound forms of tissue inhibitors of metalloproteinases, with high linearity (*r*^2^ = 0.95) for TIMP-1 and (*r*^2^ = 0.97) for TIMP-2. The intra-assay CVs that quantified variation in the assay technique itself were less than 7% for analyzed TIMPs.

### 2.4. The Assay of the Concentration of TGF-*β* in Plasma Samples

The plasma concentration of TGF-*β*1 was measured by a quantitative enzyme immunoassay technique using a specific TGF-*β*1 kit (human TGF-*β*1; BioVendor Research and Diagnostic Product, Brno, Czech Republic), according to the manufacturer's instructions. The assay detects both natural and recombinant human TGF-*β*1. All samples were measured in duplicate, and respective mean values were calculated. The limit detection of the assay was 8.6 pg/mL, and the intra-assay coefficient of variation was <4%.

### 2.5. Statistical Analysis

A statistical analysis was carried out using Statistica 12.0 package (StatSoft, Krakow, Poland). The normality of distribution was verified with the Shapiro-Wilk test. In the case of total GAGs, MMP-10, TIMP-1, and TIMP-2, the data obtained were expressed as mean values and standard deviation. Because the variables were normally distributed, the parametric Student *t*-test was used to evaluate the differences between untied variables. In the case of MMP-3 and TGF-*β*, the data obtained were expressed as medians and interquartile ranges. Since the variables were not normally distributed, the nonparametric Mann-Whitney *U* test was used to evaluate the differences between untied variables (the controls and patients). Pearson's correlation coefficient was employed for the statistical analysis of correlations between two variables. *p* values of < 0.05 were considered significant.

## 3. Results

The results are presented in Table 1. Based on the obtained results, in patients with SSc, we found a significant increase in plasma concentrations of total GAGs, quantified by the hexuronic acid assay. The patients had a 113% higher (*p* < 0.00001) level of total GAGs than the controls.

As shown in Table 1, the plasma concentrations of MMP-3 and MMP-10 in SSc are characterized by different trends of alterations. In the course of SSc, the concentration of MMP-3 in the patients' blood decreased significantly (*p* < 0.00001) when compared to this protein concentration in healthy individuals. This decrease reached 61% in MMP-3 compared with the enzyme concentration in the blood of healthy individuals. In the case of MMP-10, the concentration of the protease was similar in all groups of study individuals (*p* > 0.05).

Quantitative assessment of TIMP-1 and TIMP-2 revealed significantly higher (*p* < 0.00001) levels of these parameters in the plasma of SSc patients than in the control group. The patients had a 188% increased (*p* < 0.00001) level of TIMP-1 and a 113% increased (*p* < 0.00001) level of TIMP-2 than the controls. Moreover, a reduction in MMP/TIMP ratios was observed in SSc patients as compared to healthy subjects.

Similar trends were observed for transforming growth factor-beta concentrations (Table 1). In the group of SSc patients, TGF-*β* concentration was significantly higher (*p* < 0.0001) than in healthy individuals. When compared to the control values, the mean increase in TGF-*β* level was by 145%.

Mutual correlations of plasma GAGs and TGF-*β* levels with MMP-3, MMP-10, TIMP-1, and TIMP-2 concentrations are presented in Table 2. We recorded significant negative relationships between plasma total GAGs and TGF-*β* (*r* = −0.47, *p* < 0.05) as well as a significant positive relationship between plasma total GAGs and TIMP-2 (*r* = 0.38, *p* < 0.05) in patients with SSc. Moreover, a high concentration of TGF-*β*, recorded in patients with SSc, was negatively statistically significantly correlated with the concentrations of tissue inhibitors of metalloproteinases. The obtained values were as follows: TGF-*β* and TIMP-1 (*r* = −0.46, *p* < 0.05) as well as TGF-*β* and TIMP-2 (*r* = −0.36, *p* < 0.05), respectively. No correlation was recorded between other evaluated parameters.

**Figure 1:**
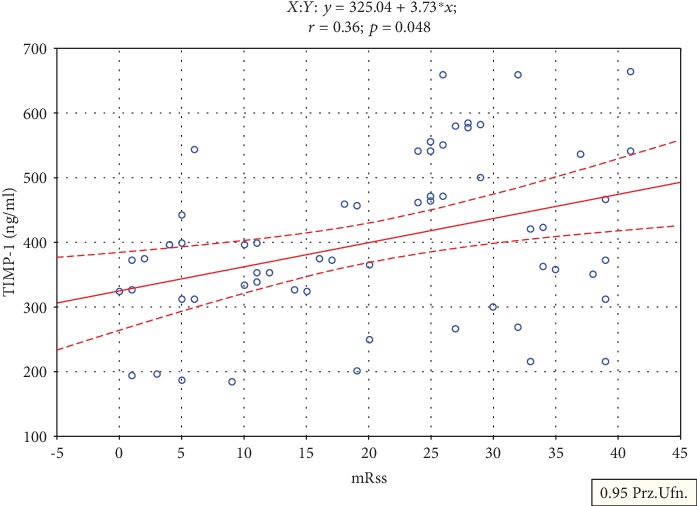
Correlation between the plasma TIMP-1 levels and the modified Rodnan skin score in patients with systemic sclerosis.

In Table 3, the correlation analysis between total GAGs, MMP-3, MMP-10, TIMP-1, TIMP-2, and TGF-*β* plasma levels and CRP, ESR, mRss, and duration of disease are presented. The conducted assessment of the relation between the abovementioned variables in SSc patients indicated a moderate positive correlation only between plasma MMP-3 concentration and duration of the disease (*r* = 0.41, *p* < 0.05) as well as plasma TIMP-1 level and mRss (*r* = 0.36, *p* < 0.05), respectively (Figure 1).

The analysis of the relations between an inflammatory indicator which is routinely assayed, i.e., CRP and MMP-3 as well as TIMP-1 concentration, revealed the positive relationship between these parameters in patients with SSc. The obtained values were as follows: CRP and plasma MMP-3 (*r* = 0.46, *p* < 0.05) and plasma TIMP-1 (*r* = 0.42, *p* < 0.05), respectively.

## 4. Discussion

Systemic sclerosis is a connective tissue disease involving fibrosis of the skin, which is characterized by vascular injury, immunological abnormalities, and excessive accumulation of extracellular matrix components, including proteoglycans and their heteropolysaccharide components, i.e., glycosaminoglycans. Previous studies evaluating GAGs in the skin obtained from people with systemic sclerosis showed an increase in the total content of these macromolecules compared to that from the skin of healthy individuals [15, 16]. Quantitative changes in PGs/GAGs occurring in the skin, and probably in other tissues and organs of patients with systemic sclerosis, manifested by excessive accumulation of these compounds, may be the result not only of increased biosynthesis but also of reduced degradation of these macromolecules. Disturbances in the metabolism of tissue proteoglycans/glycosaminoglycans in the course of scleroderma are expressed by changes in the blood plasma GAG profile, which was confirmed by the results obtained in this study. We have found that in the blood of patients with systemic sclerosis, the total concentration of glycosaminoglycans increased. The results obtained in this study coincide with the results of Fuzailova et al. [17] and Kniazeva and Khamaganova [18]. The quoted researchers have shown that the total serum GAG content increases in the course of systemic sclerosis, which is accompanied by increased excretion of these macromolecules in the urine [17, 18]. In contrast to the results described above, Hermann et al. [19] did not show any significant differences between the total plasma concentration of GAGs in the SSc patients and healthy individuals. The described discrepancy may be the result of using different analytical methods for the quantitative assessment of glycosaminoglycans.

The observed imbalance by us between the synthesis and degradation processes of tissue proteoglycans/glycosaminoglycans seems to be at the basis of the changes in the concentration of glycosaminoglycans in the blood plasma of patients with systemic sclerosis. Some authors have suggested that the accumulation in the tissues of the components in question, in the course of systemic sclerosis, is a manifestation of their increased biosynthesis [1, 16, 17, 19]. This is indicated by the results of Kuroda and Shinkai [20], obtained on the basis of the cultures of skin fibroblasts collected from the patients. Those studies revealed increased metabolic activity of pathologically affected fibroblasts. When compared to normal cells, increased synthesis of small dermatan sulfate proteoglycan, i.e., decorin, was demonstrated. SSc fibroblasts produced more both core protein and sulfated GAG chains forming these PGs. The increase in the amount of decorin synthesized by fibroblasts correlated with the increased type I collagen mRNA expression, which may indicate the participation of the described PGs in the organization of collagen fibers in the fibrosis process characteristic for SSc [20].

Despite the complexity of processes that result in enhanced PG/GAG biosynthesis in patients with systemic sclerosis, cytokines are thought to play a significant role in stimulating fibroblasts. Numerous studies have proved that TGF-*β*, PDGF, IL-4, and IL-1 (*β*) stimulate in vitro biosynthesis of PGs/GAGs by dermal fibroblasts [21–24]. However, the influence of TGF-beta on decorin biosynthesis depends on the type of cells synthesizing this PG. Thus, TGF-*β* was found to stimulate the decorin synthesis in skin fibroblasts obtained from human fetuses [22], whereas inhibitory effect of this cytokine was observed in relation to the skin and gingiva fibroblasts of the adults [21]. The inhibitory effect of TGF-*β* on the synthesis of decorin, demonstrated in experimental studies, probably also occurs in vivo. That suggestion may explain the negative relationships between the concentration of the mentioned growth factor and the concentration of total GAGs in the blood plasma of patients with SSc observed in the present study.

The reasons for the observed changes in the metabolism of proteoglycans/glycosaminoglycans in the course of scleroderma, manifested by the accumulation of these compounds in the tissues, should be seen not only in the excessive biosynthesis of these components of the extracellular matrix but also in their reduced degradation. The decreased activity of proteolytic enzymes, including matrix metalloproteinases, which degrade proteoglycan core proteins, may inhibit catabolism of tissue PGs/GAGs.

The analyses carried out as part of this study showed that among the tested metalloproteinases, MMP-3 and MMP-10, the blood plasma level in SSc patients was changed only in relation to the first of the mentioned enzyme. Found in our study is a significant decrease in plasma MMP-3 concentration which corresponded with the results of Bou-Gharios et al.'s [6] study. The cited authors observed that skin fibroblasts obtained in vitro from individuals with SSc showed a reduced MMP-3 synthesis in relation to normal fibroblasts [6].

A decrease in the concentration of MMP-3 in the blood plasma of patients with SSc observed in the present study may be the result of an increase in the activity of tissue matrix metalloproteinase inhibitors, including TIMP-1 and TIMP-2. Tissue inhibitors of metalloproteinases control matrix metalloproteinases, both by inhibiting active forms of MMPs and by inhibiting the process of their activation, i.e., transforming pro-MMP into MMPs [25]. That phenomenon seems to be reflected in our study. The results obtained in the present study showed that the mean concentration of both TIMP-1 and TIMP-2 in the blood plasma of patients with scleroderma was higher, compared to the concentrations of these inhibitors in the blood plasma of healthy individuals. The obtained results confirm the outcomes of studies by Ciechomska et al. [26], analyzing TIMP-1 concentrations, as well as the results of the studies by Yazawa et al. [27], assessing TIMP-2 concentrations. The results of the authors cited above showed that systemic sclerosis was accompanied by an increase in the concentration of metalloproteinase inhibitors in the blood [26, 27]. In turn, demonstrated in this paper, the reduction in MMP/TIMP ratios, additionally confirms the existence of a disturbed proteolytic-antyproteolytic balance favoring antyproteolytic activity. In addition, monocytes activated in SSc patients can be a source of TIMP-1, as was demonstrated by Ciechomska et al. [28]. These cells show the ability to synthesize this inhibitor after TLR8 stimulation [28].

However, the observed increase in both TIMP-1 and TIMP-2 levels in the blood plasma of SSc patients, in our study, did not correlate (*p* < 0.05) with the concentration of metalloproteinase 3. Therefore, it is assumed that the reduced concentration of MMP-3 in the blood of patients with scleroderma may be caused by the participation of other factors. Autoantibodies directed against metalloproteinase 3, present in individuals with SSc, may be one of them. These antibodies may contribute to decreased ECM degradation and consequent concentration of its components in tissues, by reducing MMP-3 concentration [3].

Among other factors, which may also regulate the concentration of MMP-3 in the blood of patients with systemic sclerosis, transforming growth factor *β* plays a significant role. It has been found that TGF-*β* inhibits collagen degradation by reducing the activity of proteinases, including MMP-1 [3, 6]. Therefore, it cannot be ruled out that the increase of TGF-*β* concentration in the blood plasma of patients with systemic sclerosis demonstrated, in this study, may be the cause of changes in the activity of other MMPs, including MMP-3. Although the results of this study did not show a direct relationship between plasma MMP-3 concentration and the concentration of the discussed cytokine, the described effect of the transforming growth factor on the decrease of the extracellular matrix of tissues cannot be excluded.

On the other hand, transforming growth factor *β* has the ability to induce the synthesis and activation of tissue matrix metalloproteinase inhibitors, which may additionally contribute to the impairment of ECM degradation [3, 6]. Therefore, the obtained results were unexpected, since they confirm an inverse relationship between the concentration of TGF-*β* and the concentration of both TIMP-1 and TIMP-2 in the blood plasma of patients with SSc. The demonstrated relationship may probably result from the simultaneous effect on these tissue inhibitors of other cytokines, growth factors, or chemokines, synthesized in excess in the course of scleroderma. Thus, overexpression of the transforming growth factor *β* stimulates the enhanced release of connective tissue growth factor (CTGF), which then can coordinate the action of TGF-*β* [29–31]. In addition, CTGF may stimulate the MMP synthesis and simultaneously inhibit TIMP-1 biosynthesis [29–31]. The above implications, explaining the contribution of many factors in metabolic disorders of the extracellular matrix components of the connective tissue, indicate the complexity of the mechanisms involved in the initiation and progression of scleroderma.

Reduced proteolytic activity observed in SSc may contribute to tissue PG/GAG accumulation, but it does not directly explain the observed increase of glycosaminoglycan concentration in the blood. Therefore, it cannot be ruled out that the increase in GAG level in the blood plasma of SSc patients is also a manifestation of increased nonenzymatic degradation of tissue PGs, stimulated by reactive oxygen species (ROS). Excessive ROS generation accompanied by a weakening of the antioxidative systems has been found as one of the pathogenic mechanisms of the SSc development [8, 31–33]. The action of these reactive molecules leads to the partial breakdown of glycosaminoglycan chains and subsequent increase in plasma GAGs level during systemic sclerosis

Although the accumulation of glycosaminoglycans in the blood could also be caused by impaired releasing of these macromolecules by the kidneys; however, in patients with SSc, there was no evidence of impaired excretion of GAGs in the urine. Furthermore, few studies found an increase in glycosaminoglycan level in the urine of SSc patients [17, 18].

The possible explanation of the observed increase in plasma GAG concentration in our study could be also related with excessive biosynthesis of endocan, the main endothelium-derived soluble dermatan sulfate proteoglycan. Increased blood level of endocan reflects endothelial activation and neovascularization, which are significant pathophysiological changes associated with inflammation accompanying systemic sclerosis [2].

The obtained results indicate that in the course of systemic sclerosis, quantitative changes in plasma glycosaminoglycans occur being most likely the expression of systemic changes in connective tissue remodeling taking part during SSc development. Quantitative determination of plasma GAG could represent a valuable marker useful in disease diagnosis; however, further studies are needed to evaluate clinical significance of this marker.

## 5. Conclusions

The results of this study revealed that remodeling of the extracellular matrix, reflected by quantitative changes in plasma glycosaminoglycans occurs during systemic sclerosis. Proteoglycan/glycosaminoglycan alterations in SSc patients, which are connected with proteolytic-antiproteolytic disturbance, indicate rather systemic disorders of extracellular matrix metabolism and not merely local changes which occur in the skin.

## Figures and Tables

**Table 1:** The distribution pattern of plasma glycosaminoglycans, plasma matrix metalloproteinases (MMP-3 and MMP-10), and their tissue inhibitors (TIMP-1, TIMP-2) as well as circulating cytokines (TGF-*β*) in the healthy controls and systemic sclerosis (SSc) patients.

Parameter	Control group (*n* = 42)	Patients with SSc (*n* = 64)
Total GAGs (hexuronic acids, mg/L)	14.24^•^ ± 4.43	30.39^•^ ± 13.87^a^
TGF-*β* (ng/mL)	14.16^∗^ (11.50–15.40)	20.64^∗^ (14.86–28.49)^b^
MMP-3 (ng/mL)	7.31^∗^ (5.86–12.04)	2.82^∗^ (2.20–5.42)^a^
MMP-10 (pg/mL)	504.35^∗^ (330.66–623.06)	662.29^∗^ (418.27–723.66)
TIMP-1 (ng/mL)	133.59^•^ ± 25.89	384.78^•^ ± 133.53^a^
TIMP-2 (ng/mL)	64.83^•^ ± 4.80	138.69^•^ ± 17.41^a^
MMP-3/TIMP-1	0.053^∗^ (0.040–0.088)	0.008^∗^ (0.005–0.015)^a^
MMP-3/TIMP-2	0.105^∗^ (0.085–0.180)	0.022^∗^ (0.015–0.042)^a^
MMP-10/TIMP-1	0.004^∗^ (0.003–0.005)	0.002^∗^ (0.001–0.002)^a^
MMP-10/TIMP-2	0.008^∗^ (0.005–0.010)	0.004^∗^ (0.003–0.005)^a^

GAGs: glycosaminoglycans; MMP: matrix metalloproteinase; TIMP: tissue inhibitor of metalloproteinase; TGF-*β*: transforming growth factor beta. ^∗^Results are expressed as medians (quartile 1–quartile 3)..^•^Results are expressed as mean ± SD. ^a^*p* < 0.00001 compared to the control group. ^b^*p* < 0.0001 compared to the control group.

**Table 2:** Correlation analysis between plasma GAGs, TGF-*β*, and MMP-3, MMP-10, TIMP-1, and TIMP-2 (Pearson's correlation coefficients, *r*).

Parameter	GAGs, *r* (*p*)	TGF-*β*, *r* (*p*)
TGF-*β*	-0.47 (0.015)	—
MMP-3	-0.24 (NS)	0.005 (NS)
MMP-10	-0.001 (NS)	-0.12 (NS)
TIMP-1	0.18 (NS)	-0.46 (0.015)
TIMP-2	0.38 (0.046)	-0.36 (0.044)

GAGs: glycosaminoglycans; MMP: matrix metalloproteinase; NS: not significant (*p* ≥ 0.05); TIMP: tissue inhibitor of metalloproteinase; TGF-*β*: transforming growth factor beta.

**Table 3:** Correlation analysis between plasma GAGs, TGF-*β*, MMP-3, MMP-10, TIMP-1, and TIMP-2 levels and duration of disease, mRss, CRP, and ESR (Pearson's correlation coefficients, *r*).

Parameter	GAGs, *r* (*p*)	TGF-*β*, *r* (*p*)	MMP-3, *r* (*p*)	MMP-10, *r* (*p*)	TIMP-1, *r* (*p*)	TIMP-2, *r* (*p*)
Duration of disease	0.18 (NS)	-0.08 (NS)	0.41 (0.023)	-0.21 (NS)	-0.23 (NS)	0.03 (NS)
mRss	0.28 (NS)	0.21 (NS)	-0.23 (NS)	0.16 (NS)	0.36 (0.048)	0.10 (NS)
CRP	0.02 (NS)	-0.20 (NS)	0.46 (0.010)	-0.04 (NS)	0.42 (0.02)	-0.04 (NS)
ESR	-0.06 (NS)	0.14 (NS)	0.13 (NS)	-0.24 (NS)	0.07 (NS)	-0.02 (NS)

CRP: C-reactive protein; ESR: erythrocyte sedimentation rate; GAGs: glycosaminoglycans; MMP: matrix metalloproteinase; mRss: modified Rodnan skin score; NS: not significant (*p* ≥ 0.05); TIMP: tissue inhibitor of metalloproteinase; TGF-*β*: transforming growth factor beta.

## Data Availability

The data used to support the findings of this study are available from the corresponding author upon request.
